# Detecting fake news and disinformation using artificial intelligence and machine learning to avoid supply chain disruptions

**DOI:** 10.1007/s10479-022-05015-5

**Published:** 2022-11-01

**Authors:** Pervaiz Akhtar, Arsalan Mujahid Ghouri, Haseeb Ur Rehman Khan, Mirza Amin ul Haq, Usama Awan, Nadia Zahoor, Zaheer Khan, Aniqa Ashraf

**Affiliations:** 1 University of Aberdeen Business School, University of Aberdeen, King’s College, AB24 5UA Aberdeen, UK; 2 Imperial College London, SW7 2BU London, UK; 3 Faculty of Management and Economics, Universiti Pendidikan Sultan Idris, Tanjong Malim, Malaysia; 4 Faculty of Art, Computing, and Creative Industry, Universiti Pendidikan Sultan Idris, Tanjong Malim, Malaysia; 5 Department of Business Administration, Iqra University, Karachi, Pakistan; 6 Department of Business Administration, Inland School of Business and Social Sciences, Inland Norway University of Applied Sciences, Hamar, Norway; 7 School of Business and Management, Queen Mary University of London, London, UK; 8 CAS-Key Laboratory of Crust-Mantle Materials and the Environments, School of Earth and Space Sciences, University of Science and Technology of China, 230026 Hefei, PR China; 9 Innolab, University of Vaasa, Vaasa, Finland

**Keywords:** Fake news, Disinformation, Misinformation, Artificial intelligence, Machine learning, Supply chain disruptions, Effective decision making

## Abstract

Fake news and disinformation (FNaD) are increasingly being circulated through various online and social networking platforms, causing widespread disruptions and influencing decision-making perceptions. Despite the growing importance of detecting fake news in politics, relatively limited research efforts have been made to develop artificial intelligence (AI) and machine learning (ML) oriented FNaD detection models suited to minimize supply chain disruptions (SCDs). Using a combination of AI and ML, and case studies based on data collected from Indonesia, Malaysia, and Pakistan, we developed a FNaD detection model aimed at preventing SCDs. This model based on multiple data sources has shown evidence of its effectiveness in managerial decision-making. Our study further contributes to the supply chain and AI-ML literature, provides practical insights, and points to future research directions.

## Introduction

The increased scholarly focus has been directed to fake news detection given their widespread impact on supply chain disruptions, as was the case with the COVID-19 vaccine. Fake news and misinformation are highly disruptive, which create uncertainty and disruptions not only in society but also in business operations. Fake news and disinformation-related problems are exacerbated due to the rise of social media sites. Regarding this, using artificial intelligence (AI) to counteract the spread of false information is vital in acting against disruptive effects (Gupta et al., 2021). It has been observed that fake news and disinformation (FNaD) harm supply chains and make their operation unsustainable (Churchill, 2018). According to research, fake news can be classified into two distinct concepts of misinformation and disinformation (Petratos, 2021; Allcott & Gentzkow, 2017) defined fake news as “*news articles that are intentionally and verifiably false, and could mislead readers*” (p. 213). According to Wardle (2017), misinformation refers to “*the inadvertent sharing of false information*”, while disinformation can be defined as “*the deliberate creation and sharing of information known to be false*”. Among the negative consequences that fake news can have for companies are loss of sponsorships, reduced credibility, and loss of reputation which can adversely affect performance (Di Domenico et al., 2021). In such a context AI is shaping decision-making in an increasing range of sectors and could be used to improve the effectiveness of fake news timely detection and identification (Gupta et al., 2021). Whereas many new efforts to develop AI-based fake news detection systems have concentrated on the political process, the consequences of FNaD on supply chain operations have been relatively underexplored (Gupta et al., 2021).

Kaplan and Haenlein (2019) addressed AI “*as a system’s ability to interpret external data correctly, to learn from such data, and to use those learnings to achieve specific goals and tasks through flexible adaptation*” (p.17). Although emerging technologies such as AI may sometimes have negative effects, they can be utilized to combat disinformation. As scholarship is showing increasing interest in how AI can improve operationally and supply chain efficiencies (Brock & von Wangenheim, 2019), researchers have recently called for more studies on how organizational strengths and the use of AI influence the outcomes for decision-making structures (Shrestha et al., 2019). Fake news has considerable negative effects on firms’ operations, such as repeated disruptions of supply chains (Churchill, 2018). FNaD influence the use of a company’s product or services (Zhang et al., 2019; Sohrabpour et al., 2021) argued that leveraging AI to improve supply chain operations will likely improve firms’ planning, strategy, marketing, logistics, warehousing, and resource management in the presence of any environmental uncertainty, including that caused by FNaD.

Scholars have called for research to attain an in-depth understanding of AI and of how to tailor it to enhance business efficiencies and minimize supply chain disruptions (SCDs) (e.g., Grewal et al., 2021; Churchill, 2018). The extant literature has drawn mixed conclusions on whether AI-driven or hybrid AI decision-making benefits a firm’s supply chain (Shrestha et al., 2019). The question of why some firms are more effective than others in using AI to manage SCDs has largely been overlooked (Toorajipour et al., 2021). Increased research efforts are being made to identify and manage fake news risk in supply chain operations (Reisach, 2021). In today’s digital media landscape, the term ‘fake news’ has gained relevance following the 2016 US presidential elections (Allcott & Gentzkow, 2017). People have been observed to be unable to clearly distinguish between fake and real news and to tend to perceive ‘fake news’ as a more significant issue within the current information landscape (Tong et al., 2020). Therefore, decision-makers are often influenced by FNaD, thus ending up making erroneous decisions and drawing inaccurate conclusions regarding current scenarios (e.g., Lewandowsky et al., 2012; Di Domenico et al., 2021). From a supply chain perspective, researchers have highlighted how FNaD can lead to SCDs (e.g., Gupta et al., 2021; Kovács & Sigala, 2021; Sodhi & Tang, 2021), which can have a far-reaching impact on the functioning of global supply chains.

Additionally, the United Nations (2020) have suggested that, despite the measures put in place to build confidence in people, businesses, and supply chain operations, SCDs have remained a problematic area for businesses in recent years. Resilinc (2021) revealed that SCDs have been increasing by 67% year-over-year, with 83% of such disruptive events being caused by human activity—not natural disasters. EverStream Analytics (2020) found that 40.5% and 33.4% of businesses are respectively getting their information and intelligence relating to supply chain issues from their customers and social media. The detection of fraudulent information is thus critical to avoid such consequences (Kim & Ko, 2021), and businesses need to set up specific processes or routines to filter incoming business-related information and mitigate any possible related harm to their operations (Kim & Ko, 2021; Kim & Dennis, 2019) emphasized research underpinning emerging technologies such as AI suited to tackle FNaD. As FNaD have become increasingly relevant in the field of operations management, and given their effects on decision-making, there is a need to understand what business processes require to be implemented to contain their spread and minimize SCDs.

However, there is still a limited understanding of how AI techniques can help in eliminating FNaD. We, therefore, sought to define an AI-oriented business process suited to remove the effects of FNaD on decision-making and set our research question as: “*How can firms integrate AI in their operations to reduce the impact of FNaD regarding SCDs*?” In answering this question, our study makes three contributions to the literature. First, it develops a new theoretical framework suited to mitigate the impacts of FNaD on SCDs and it analyses the relationship using a specific dataset and support-vector machine. The resulting business process manages the dissemination of information, accurately mitigating FNaD and enabling correct decision-making in regard to tackling complex issues (e.g., Jayawickrama et al., 2019). Second, by presenting key findings gleaned by interviewing senior managers from three different countries (Indonesia, Malaysia, and Pakistan) with expertise in supply chains, our study provides new theoretical evidence regarding how firms can avoid SCDs in emerging economies. To the best of our knowledge, our study is the first to focus on the implications and integration of AI in business processes to the end of mitigating the effects of FNaD on SCDs. Our framework thus links the supply chain and AI literature and explicates their utility in mitigating SCDs against the backdrop of fake news and disinformation campaigns. In our study, we adopted a qualitative method that involved integrating the AI literature with research on fake news to reveal how the effectiveness of decision-making can be ensured within supply chain operations. Much previous research has advanced our understanding of fake news detection mechanisms using graphs and summarization techniques (Kim & Ko, 2021). Furthermore, a recent study has proposed an AI-based, real-time fake news detection system by conducting a systematic literature review (Gupta et al., 2021). Third, our study fills a gap in the literature by providing a practical solution aimed at eliminating or reducing FNaD in business scenarios, specifically acting to minimize SCDs. The extant literature is somewhat scattered and fragmented that has not helped researchers to address many questions about FNaD (Di Domenico & Visentin, 2020). Our study proposes an AI-oriented business process that flags/reduces/eliminates FNaD before it can reach decision-makers and allows authentic news and information to filter through to supply chain operation resilience and prevent SCDs.

This paper is structured as follows. Section2 presents a discussion of the related literature, which is followed by an illustration of our research methodology in Sect. 3. In Sect. 4, the implementation details, findings, and proposed model are provided. In Sect. 5, the implications of our model are discussed and, to conclude, future research directions are suggested.

## Literature review

### Theoretical background

Organizational Information Processing Theory (OIPT) proposes a systematic comprehension of processing and exchanging of information to increase capacities. OIPT reasons that firms need a stabilizing mechanism by possessing resources and capacities in operations to cope with uncertainties and manage unforeseen events that disturb normal business and supply chain operations (Wong et al., 2020). Scholarship suggests that SCDs could be caused by disinformation (e.g., Konstantakis et al., 2022; Xu et al., 2020). It is ultimately inevitable for supply chains to cultivate the capability and capacity to proactively engage the filtration of the information and news to improve supply chain operations. Firms could either opt to rely on mechanistic organizational resources for reducing their reliance on information or enhance their information processing capabilities. The more environmental uncertainty facing firms, the more information they need to gather and process to achieve better performance (Bode et al., 2011). OIPT proposing the primary goal of organizational-related process designs is linked with uncertainty by acquiring, analyzing, and sharing information from the business environment (Swink & Schoenherr, 2015; Yu et al., 2019). OIPT addresses the development of organizational capabilities to fill their information processing requirements (Wamba et al., 2020). SCDs can be avoided by the filtration of receiving accurate and timely information. Di Domenico et al., (2021) suggested that FNaD during disruptions i.e., the supply chain may cause the loss of preventable lives, misguiding information on business activities and innovation. Fact-checking measures like “know why”, “know how”, “know what”, and “know when” could be checked by emerging technologies and information processing capabilities (Jayawickrama et al., 2016; Swanson & Wang, 2005). In this perspective, AI and Machine Learning (ML) could manage the dissemination of real information by accurately detecting and mitigating false information and making correct decisions when tackling difficult issues (Endsley, 2018; Jayawickrama et al., 2019; Roozenbeek and van der Linden, 2019). OIPT thus focuses on linking uncertainty with information needs and information processing capacities and prescribes organizational designs to reduce uncertainty. Our study thus seeks to provide a holistic theoretical framework (integrated with AI and ML) built based on OIPT to minimize the chances of SCDs.

### Artificial intelligence and supply chain operations

In academia, the concept of AI was first established in the 1950s (Haenlein & Kaplan, 2019). However, McCulloch & Pitts (1943) ideas on logical expression represent a notable landmark, as they led to the development of a neurocomputer design (Milner, 2003). While the exact year is unknown, the origins of AI can thus be dated to the 1940s; notably, to Isaac Asimov’s 1942 short tale ‘Runaround’, published in ‘Science Fiction’ magazine. In it, Asimov formalized his three laws of robotics: first, a robot cannot harm a human being; second, a robot must follow human commands; and third, a robot must defend itself (Haenlein & Kaplan, 2019). In 1955, in a research project on AI (McCarthy et al., 1955) Dartmouth college defined it as “*making a machine behave in ways that would be called intelligent if a human were so behaving*” (p.11). Since 1955, AI has evoked the idea of relevant human intuition and artificial machines that could stimulate the human brain and come up with environmental abstractions to work on difficult problems. During the following decade, in 1966, Joseph Weizenbaum created the famous ELIZA computer program, a ‘natural language processing (NLP) tool that was capable of holding a conversation with a human being and maintaining the illusion of comprehension. This was labelled heuristic programming and AI (Weizenbaum, 1966). In the 1980s, research on backpropagation in neural networks saw rapid development (Zhang & Lu, 2021). Under Ernst Dickmanns, Mercedes-Benz developed and commercialized a driverless vehicle fitted with cameras and sensors and an onboard computer system controlling the steering (Delcker, 2018). With the continuous development of AI tools, the success of IBM’s ‘Deep Blue’ chess-playing supercomputer laid the foundations for research on and the application of expert systems (Haenlein & Kaplan, 2019).

AI is viewed as a game-changer and as being able to facilitate both the “*abilities to self learn and a race to improve decision quality*” (Vincent, 2021, p. 425). Kaplan and Haenlein (2019) defined AI “*as a system’s ability to interpret external data correctly, to learn from such data, and to use those learnings to achieve specific goals and tasks through flexible adaptation*” (p.17). In supply chain management and the manufacturing industry, there has been an upsurge in AI (Kumar et al., 2019) that has significantly impacted operations and human roles in firms (Vincent, 2021; Awan et al., 2021) suggested that AI initiatives in firm supply chain operations can improve knowledge of the processes used to generate business performance. AI is a complex and multifaceted construct with profound implications for firm operations management (Zeba et al., 2021). The supply chain literature has recently emphasized the link between the application of AI and process improvement (Toorajipour et al., 2021). Although several AI-based supply chain applications have appeared in recent years, little research has explored their use (Riahi et al., 2021). While the debate on the operational outcomes of AI is still ongoing, there is little evidence in the operations management literature of how the adoption of AI may improve supply chain operations (Raisch & Krakowski, 2020).

Recent advancements in material and production technologies hold great possibilities for a better understanding of how to improve other manufacturing and supply chain operations (Grewal et al., 2021). AI-based models provide near-optimal solutions to a wide variety of routing challenges, ensuring on-time deliveries and optimizing warehouse transport (Riahi et al., 2021). However, little attention has been devoted to how the use of AI techniques may affect the reverse auctioning that involves supply chain partners and planning for vehicle routing and volume discount acquisition (Toorajipour et al., 2021). By affecting decision-making and increasing effective knowledge creation aimed at developing products customized for specific situations, AI technologies may have significant implications for a firm’s production capabilities (Awan et al., 2021). As a creative and frequently disruptive technology, AI facilitates the design of new products, services, industrial processes, and organizational structures that meet client needs. Further, product, service, manufacturing, or organizational processes can be designed using AI (Wamba-Taguimdje et al., 2020). For B2B companies, customer understanding is critical to boost products or services (Paschen et al., 2019). The integration of AI with the industrial Internet of Things holds significant potential for solving production-process problems and making better-informed decisions (Zeba et al., 2021). Early adopters of AI have created new and improved goods, which has enabled them to outperform the competition (Behl et al., 2021). By analyzing market intelligence, AI can uncover themes and patterns in data and may provide insights into how users creatively alter products and services (Paschen et al., 2019). A growing number of scholars are maximizing the influence of AI on supply chain risk management and monitoring systems to avoid SCDs (Toorajipour et al., 2021). However, little is known about its role in shaping monitoring, and controlling supply chain operations (Pournader et al., 2021). Although research has found that AI is used to improve supply chain performance, just a few AI approaches and algorithms have been explored and are used in supply chain processes (Riahi et al., 2021).

AI is linked to analytical, self-learning, and predictive machine learning approaches (Shrestha et al., 2019). These methods offer a variety of answers and prescriptive inputs to choose from when deciding how to proceed with complicated scenarios (Belhadi et al., 2021). Even though researchers have focused on the use of AI in different fields of study, it is important to note that very few studies have looked at how AI can be used in enhancing supply chain operations. However, the importance of AI in predicting and mitigating supply chain risk has been well established in the literature (Riahi et al., 2021). AI can accurately and rapidly detect relevant supply chain information by using analytics produced through AI techniques and models. They give managers a greater understanding of how each system operates and help them to discover areas in which they can improve those operations. The development of AI has made it possible to deploy predictive algorithms that allow for faster evaluations and more effective risk minimization across supply chains (Ni et al., 2020). The extant literature on AI argues that applying different machine learning approaches with AI can substantially decrease SCDs (Riahi et al., 2021). AI and ML enhance operations in many domains, including supply chain management, logistics, and inventory management (Belhadi et al., 2021; Ni et al., 2020) showed that supply chain managers can use AI to watch for and avoid incidents interrupting supply chain operations. This includes everything from the most prevalent occurrences to unknown factors such as delivery delays, quality defects, among others (Belhadi et al., 2021).

AI provides the opportunities and promises to move toward data-driven decision support systems. Despite the integration of AI in many firm processes, there are still challenges regarding the design of a firm supply chain that depends heavily on human contributions (Kumar et al., 2019). However, it has been established in the operations management (OM) literature that AI has a positive impact on various supply chain management activities (Dubey et al., 2021). Still, it rarely addresses how AI is applied in the OM field, such as in manufacturing, production, warehousing and logistics, and robot dynamics (Toorajipour et al., 2021). Even though the supply chain literature has acknowledged that many AI applications include production forecasting, supplier selection, material consumption forecasting, and customer segmentation (Toorajipour et al., 2021), the AI literature typically revolves around understanding effective ways to combine human intuition and decision-making (Vincent, 2021). The use of AI technologies gives marketers a competitive edge that reflects marketing tactics and customer behaviors (Jabbar et al., 2020). Customer order processing can be automated with AI, and chatbots can handle any follow-up chores (Paschen et al., 2019), which can increase supply chain effectiveness. It is possible to take proactive measures to combat supply chain risks by uncovering new trends in the data; this is expected to assist in achieving adaptability and higher levels of supply chain maturity (Riahi et al., 2021). Multiple courses of action are open to firms confronted with the risk linked to investing in AI and its positive impacts on supply chain activities. The proliferation of evolving AI technology has led to premature and conflicting conclusions regarding specific outcomes.

Scholars increasingly recognize the importance of AI in lowering downtime costs, better utilizing real-time data, better scheduling, and preserving firm operations from risks (Chen et al., 2021). Additionally, Chen et al., (2021) suggested a predictive maintenance framework for the management of assets under pandemic conditions, including new technologies, such as AI, for pandemic preparedness and the avoidance of business disruptions. The implementation of AI-based systems influences supply chain inventory management, “*for instance performance analysis, resilience analysis or demand forecasting*” (Riahi et al., 2021, p.13). This raises the question of whether the use of AI systems to determine short-order policies and mitigate any bullwhip effects has been adequately addressed in the literature (Preil & Krapp, 2021). A review found that the adoption of AI in supply chains improves performance, lowers costs, minimizes losses, and makes such chains more flexible, agile, and robust (Riahi et al., 2021). Recent advances in AI help supply chain firms to enhance their analytics capabilities, leading to improved operational performance (Dubey et al., 2021). AI-enabled supply chain performance is becoming increasingly important to enhance financial performance; yet, no studies have been hitherto conducted to the end of gaining a better understanding of the critical antecedents of AI in driving supply chain analytics (Dubey et al., 2021). The direct and positive impact of AI-based relational decision-making on firm performance has been established (e.g., Bag et al., 2021; Behl et al., 2021).

### Fake news, disinformation, and supply chain disruptions

Fake news (Oxford English Dictionary, 2021a) is defined as: “*false reports of events, written and read on websites.*” Furthermore, disinformation (Oxford English Dictionary, 2021b) is construed as: “*false information that is given deliberately*”. The impact of FNaD is substantial, disrupting economic operations and societal activities. FNaD also threaten brand names and potentially affect the consumption of products and services, ultimately impacting supply chain operations and demand (Zhang et al., 2019; Petratos, 2021). These are affected by panic-driven or bad decisions based on disinformation (e.g. Ahmad et al., 2019; Matheus et al., 2020; Zheng et al., 2021). SCD is defined as a disturbance in the flows of material, financial, and information resources between firms and their major stakeholders—e.g., suppliers, manufacturers, distributors, retailers, and customers. Disruption may affect supply chain operations for random periods (Mehrotra & Schmidt, 2021). Supply chains encompass the activities needed for firms to deliver products and services to their final consumers, and accurate information is an integral part of such chains, as it enables decision-makers to make decisions on future demand, supply, cash flows, returns, among other supply chain operations. There are historical examples of how FNaD can affect the supply chain and business operations. From the 1950s to 1990, the tobacco industry constantly shared disinformation on the adverse effects of active and secondhand smoke exposure by manipulating research, data, and the media (Bero, 2005; Dearlove et al., 2002). In September 2006, the Royal Society, Britain’s premier scientific academy, wrote to ExxonMobil urging it to stop funding the dozens of groups spreading disinformation on global warming and claiming that the global temperature rise was not related to increases in carbon dioxide levels in the atmosphere (Adam, 2006). In 2013, the Associated Press official Twitter account was hacked and a tweet was made about two explosions injuring President Barack Obama; within hours, this wiped US$130billion from the stock market (Parsons, 2020; Tandoc et al., 2018), which affected stock supply chain operations. In 2017, six UK Indian restaurants fell victim to fake news stories claiming that they were serving human flesh (Barns, 2017; Mccallum, 2017). One restaurant had to cut staff hours and saw its revenue fall by half (National Crime Agency, 2018). Such events could also have indirect effects on supply chain operations, ultimately being conducive to SCDs.

The persuasive power of fake news could continuously damage global supply chains, such as meat, vegetables, fresh food, and fruits in different parts of the world (Xu et al., 2020). All businesses have felt the rapid dissemination of false information and propaganda among suppliers and distributors. Many countries such as France, Germany, India, and the US imposed restrictions on products from entering and leaving different countries due to disinformation and fake news about Covid-19 (Xu et al., 2020). Moreover, several fake social media posts or misinformation about the U.S. food plant fire caused the food supply disruption. The USDA told Reuters via email that it is not true that these fires were started on purpose (Reuters, 2022). Due to the widespread false information about COVID-19, there was an epidemic of methanol poisoning. It is claimed that 796 Iranians lost their lives to the alcohol intoxication after reading online claims that alcohol may treat their illnesses (Mahdavi et al., 2021). This echoed the rapid dissemination of false information regarding COVID-19 on social media at the outbreak’s onset and disrupted the supply of many food items (Mahdavi et al., 2021). Some people falsely claim that using alcohol to rinse the mouth and avoid COVID-19 infection works (Delirrad & Mohammadi, 2020; Soltaninejad, 2020). The global supply chains have been shaken by the widespread of fake news causing widespread disruptions and affecting firms’ reputations. The effects of fake news on COVID-19 are still being felt by supply chains across many sectors, and it has irrevocably affected long-term supply chain strategies.

In more recent times, the COVID-19 pandemic has spawned high volumes of FNaD. One example of disinformation pertaining to a COVID-19 remedy involved a herb named ‘*Senna Makki*’ in Pakistan., Someone started sharing on social media as a cure for the virus, which caused escalating demand and an increase in price from 1.71USD to 8.57-11.43USD per kg within two months (The News, 2020). This kind of fake news could affect vaccine supply chains. In the stock market, Clarke et al., (2020) revealed that a well-known crowd-sourced content service for financial market websites had been generating fake news stories due to the editors’ lack of ability to detect them. This attempted use of fake news had ‘widespread short-term implications for the financial markets. The Kroll global fraud and risk report of 2019/20 shared an incident of fake news in the banking industry. The rival institution purchased an African bank, the purchaser was confronted with a negative social media campaign, fabricated news, and stories, and manipulated closed-circuit television footage (Booth et al., 2019). These examples demonstrate how FNaD can disrupt supply chains and business operations.

Our review of past studies yielded Table 1, which summarizes key studies interlinking AI, SCDs, and FNaD. These studies are selected from top-ranked journals (e.g., CABS 3 ranked and above) published in the last three years. The lack of research on all three aspects is very clear, with no studies emphasizing their combination. Our study significantly contributes to bridging this gap.

**Table 1:** Selective studies on AI and supply chain operations

Author, year, journals ^a^	Key Points	Research gap	AI + SCDs + FNaD
(Behl et al., 2021) (AOR)	AI, operational efficiency, trust, and transparency	How does a firm establish links between AI, SCM, and risk management?	Enabling artificial intelligence by limiting erroneous information.
(Chen et al., 2021) (AOR)	Proactive maintenance and human-centered AI decision system.	What is the impact of Trust in AI on human-centric decision support	AI’s application for predictive maintenance to reduce uncertainty.
(Bag et al., 2021) (IMM)	AI, relationship management, and firm performance	The use of AI is a prerequisite for market knowledge creation.	Artificial intelligence Fake news and relationship management.
(Dubey et al., 2021) (IMM)	Alliance management, AI, and pandemic crises	The examination of the key antecedents of AI, as driven by Supply Chain Analytics	AI-driven supply chain firms’ internal dynamic capabilities lower the SCDs.
(Grewal et al., 2021) (JBR)	The dark side of AI Does it make procurement more efficient?	How can AI improve fraud detection in SC?	AI is expected to minimize the SCDs caused by uncertain external events.
(Mikalef et al., 2021) (IMM)	AI capability, uncertain environment, and firm performance.	The examination of the antecedents of developing resources inside your own company	AI can improve the decision-making process, and Lowering risk facilitates high-value analysis.
(Pournader et al., 2021) (IJPE)	AI, operational performance, and Information Management	The examination of the relationship between MIS and human behavior in SCM	AI can gain SC operational performance in the face of information uncertainty.
(Zeba et al., 2021) (TFSC)	AI technologies production systems.	AI technologies production systems and leveraging knowledge on firm sustainability performance.	AI supports knowledge protection and minimizes the risk of disinformation to do so.
(Farrokhi et al., 2020) (IMM)	AI, crisis management, and decision-making.	The use of AI to explain business situations is still evolving.	AI can be used to detect disinformation and misinformation.
(Shrestha et al., 2019) (CMR)	AI trust and developing internal capabilities.	How is the hybrid human-AI decision-making process beneficial?	AI could be useful for accurate decision-making regarding disinformation and the reduction of risk.

*Note*: ^*a*^*(AOR): “Annals of Operations Research, (CMR): “California Management Review, (IMM): “Industrial Marketing Management”, (IJPE): “International Journal of Production Economics, (JBR); “Journal of Business Research”, (TFSC); “Technological Forecasting & Social Change”*

## Methodology

We used mixed methods, the AI and ML-driven method, and our case study interviews to further validate our model. The following procedure was used to execute the AI and ML-driven method for data analysis. (1) The Dataset Enrichment was based on two techniques—i.e., Porter stemmer (PS) and Term Frequency-Inverse Document Frequency (TFIDF). (2) Query Expansion was utilized for natural language processing (NLP) to precisely predict the accuracy of fake news. (3) The Support Vector Machine (SVM) classifier was utilized to train the model and then finally evaluate fake and real news outcomes for effective decision-making SCDs. Table 2 provides further justification for this approach. The studies also used other measures such as precision, recall, and accuracy and we also integrated similar measures in our analysis.

**Table 2:** Algorithms and approaches used by previous studies for FNaD detection

Study	Application	Approach/classifiers/algorithm
Jiang et al., (2021)	Fake news detection	SVM Logistic Regression Decision Tree and others Random Forest TFIDF
Wang et al., (2020)	Fake News Detection	SVM Binary Classifier
Zhou et al., (2020)	Early fake news detection	SVM Logistic Regression Random Forests and others
Kareem & Awan (2019)	Fake news classification	SVM K-Nearest Neighbor Random Forest and others PS
Ibrishimova & Li (2018; 2019)	Fake news detection	Binary Classifier TensorFlow’s Linear Classifier and others Semantic similarity measures and WordNet ontology
Poddar & Umadevi (2019)	Accurate fake news detection	Support Vector Machine (SVM) Logistic Regression Decision Tree Artificial Neural Networks
Sabeeh et al., (2019)	Enhanced fake news detection	SVM Decision Tree
*FNaD (our study)*	*Fake news and disinformation*	*SVM* *PS* *TDIF* *Semantic similarity measures & WordNet*

Secondly, the involved cases for interviews were from Indonesia, Malaysia, and Pakistan. These countries share similarities. Furthermore, these are emerging countries with common economic and political ties as well as government-to-government contacts. Similarly, these nations are making strides toward digitization and AI. Numerous previous studies (e.g., Atkin et al., 2017; Ghazali et al., 2018; Rahi et al., 2019; Siew et al., 2020) also used targeted populations from these countries to conduct studies on comparable issues. The case study method was best suited to achieve the objectives of our study, considering the explanatory nature of the research question (Eisenhardt & Graebner, 2007; Yin, 2014) and the fact that this is an emergent research area to be combined with modern methodological innovations such as ML (Gupta et al., 2021; Kovács & Sigala, 2021; Sodhi & Tang, 2021; Sheng et al., 2020). Further, multiple case studies are assumed to be more reliable because they enable phenomena to be observed and studied in many contexts—thereby helping to provide replication logic for particular cases, which would otherwise be viewed as independent (Yin, 2014)—and because they are useful for theory development (Eisenhardt, 1989).

A goal-directed sampling technique—i.e., an incremental selection method (Denzin & Lincoln, 2005)—was employed to investigate the flagging/reducing/eliminating process of FNaD. This technique is effective for the collection of both qualitative and quantitative data because the sample is purposely chosen based on the project’s unique requirements and the evaluator’s judgment (Polit & Beck, 2012; Vos et al., 2011). The data acquired using this approach and technique tend to be of a high standard only if the participants are willing and able to provide accurate information that will enable the researcher to gain a thorough knowledge of an experience. We thus adopted this sampling technique because our target key management functionaries would be the participants best suited to furnish the essential data for our study (Creswell, 2014), providing useful insight into what works and what does not in terms of its theoretical and technical components. Any case variations were scrutinized based on the industry to achieve a better understanding of FNaD impacts.

To conduct the interviews, we approached 33 firms—36.36% Malaysian, 36.36% Pakistani, and 27.28% Indonesian ones. Eventually, a total of 16 firm representatives participated in our study—six each from Malaysia and Pakistan, and four from Indonesia. According to Teddlie & Yu (2007), this was a sample size sufficient to produce narrative records adequate to provide viewpoints directly relevant to the topic under study. The sample firms were small and medium-sized, with staff numbers ranging from 18 to 183. Small businesses have learning systems that are complex and dynamic and geared to generate the efficiency needed to sustain such firms in the market (Zhang et al., 2006). Therefore, an in-depth investigation was needed to understand the stances of small and medium-sized firms about FNaD. Fifteen semi-structured in-depth interviews were prepared, based on the interviewees’ understanding of their respective firms and the inflow and outflow information features such as sources, routines, systems, and processes. The interviewees were owners, chief executive officers, directors, and associated top managers. The participants’ varied perspectives reduced dependency on a single participant’s perspective, enriching the data obtained. We also used project reports, operational policies, and other relevant documentation to identify and triangulate themes during the data analysis (Yin, 2014). Due to the COVID-19 pandemic, 90% of the interviews were conducted over the internet (e.g., GoogleMeet) and any observations were recorded and reviewed later.

We followed the interview protocol to integrate the philosophy, processes, and questions of the study to attain reliability (Frost et al., 2020; Ponterotto, 2005). To delve deep into the situation about the impacts of fake news and any remedial actions, we used relevant prompts in open-ended questions. The extensive conversations provided key findings regarding the solutions adopted to counter the effects of fake news on business and supply chain operations by reflecting on three industry and technology developments, gathering din depth and valuable narratives in the process. All interviews were audio-recorded, converted into MSWORD, and thematically analyzed via NVIVO 12. To ensure validity and reliability, each researcher independently coded the responses given to the open-ended questions to fully grasp any concepts that were not readily provided by existing theories or field research. The answers were discreetly coded to fully understand any new sentiments, knowledge, and opinions that may not be available in the literature on the selected industries and countries. This practice provided an AI-based solution to fake news issues in business. Furthermore, other consistency checks were carried out, whereby the data and preliminary interpretations were presented to the interviewees from whom they had been sourced to determine their credibility and incorporate any necessary changes, and the scripts were then finalized following their approval (Merriam, 2009).

## Implementation procedures, findings, and proposed model

### AI and ML implementation

#### Obtaining the dataset

The dataset for the AI and ML approach was drawn from four Pakistani major online news sources—i.e., ‘Geo News’, ‘The Dawn’, ‘Express Tribune’, and ‘The News’. Approximately 500 pages from each source were scrutinized to extract the relevant affairs and topics from January to April 2021. SCD data were divided into natural, human-caused, maritime, and mass disruptions related to FNaD. Table 3 provides some examples.

**Table 3:** Examples of SCD-related words/data

Natural	Human-caused	Maritime	Mass
“Tornadoes and Severe Storms” “Hurricanes” “Tropical Storms” “Floods” “Wildfires” “Earthquakes” “Drought”	“Industrial accidents” “Shootings” “Acts of terrorism” “Mass Labor strikes” “Incidents of mass violence” “Nuclear Facilities failure” “Political Crises” “Wars”	“Offshore Oil Rig Mishaps” “Cruise Vessel Mishaps” “Commercial Fishing Mishaps” “Accidents on Tugboats” “Accidents on Crude Oil” “ Tankers and Cargo Ships” “Grounding of Ships” “Crane Mishaps” “Accidents in Shipyards” “Cargo Hauling Accidents” “Port Delays”	“Infectious disease outbreaks” “Incidents of community unrest” “Mass migration and refugees” “Covid-19” “Pandemic”

The words were used in different contexts. For example, Words such as health, vaccine, Covid-19, and pandemic were mostly used in the main text in articles related to health supply chains and their disruptions, and words like political, freedom, rights, democracy, and military were applied in political articles. We followed a step-by-step procedure for the implementation.

#### Pre-processing dataset

• PS (Porter Stemmer) was used to index each news page/article to filter out any stop, repeated, and common words to avoid noise in the dataset. The algorithm was used, over several rounds, to remove any non-relevant words from the datasets/textual scripts before considering all criteria or defined rules (Zhang et al., 2020). Such an algorithm has been proven to be one of the best techniques in terms of performance (Joshi et al., 2016).
• TDIF (Term Frequency - Inverse Document Frequency) was utilized for the classical ML models. TDIF is a text classification technique utilized for organizing textual documents from raw datasets into predefined categories to obtain useful information. This is done by representing textual documents into feature vectors consisting of weights that indicate the contribution of each term in text classification (Deng et al., 2004; Dogan & Uysal, 2019). The effectiveness of TDIF has also been proven to be significant in the weighting process (Dogan & Uysal, 2019).

#### Dimension reduction and features engineering

The Query Expansion.

In the field of natural language processing (NLP) and information retrieval such as metrics of text semantic similarity are the most used techniques (Zhu et al., 2018, 2020; Gao et al., 2015). The query expansion approach was utilized for natural language processing to precisely compute the relevancy of keywords related to FNaD by calculating the semantic distance between keywords related to SCDs. This phase explained the keywords used in SCDs to train the classifier to predict the appropriateness of SCDs in the news. As a feature, we analyzed the significance of each SCD keyword to a news category. We had the news articles in the business category and we wanted to evaluate whether it is appropriate for SCDs. The degree of appropriateness was based on the feature’s relevancy score; +1 perfectly appropriate and − 1 vice versa. The outputs of fake or real were decided based on the scores. Each keyword pair of news category and supply chain disruptions were considered appropriate if the relevancy score is + 0.65. As a result, we trained the SCD keywords appropriateness to predict based on the remaining dataset. We trained various frequently used classifiers and reported SVM results as SVM outperformed others.

Query Expansion: The query expansion approach was utilized for natural language processing (NLP) to precisely compute the relevancy of keywords related to FNaD by calculating the semantic distance between keywords related to SCDs, queries, and news articles. This phase explains the keywords used in SCDs to train a classifier to predict the appropriateness of SCDs in the news. We analyze the significance of each SCD keyword to a news category. We have news articles in the category “Business” and we wish to evaluate whether it is appropriate for a SCD example such keywords in the category; “natural disasters”, “man-made disasters”, “marine incidents”, and “mass trauma incidents”. We consider the degree of appropriateness of the news category with each of the SCD keywords based on the feature’s relevancy score. In this scenario, -1 represents that a news item with the category “sports” is utterly inappropriate to be examined with supply chain disruption keywords “offshore oil rig mishaps,“ but a + 1 score suggests that it is perfectly appropriate. Table 4 shows the examples of the relevancy score of the news category against supply chain disruption keywords present in our database.

**Table 4:** Example of supply chain disruption features

Category (News)	Supply Chain Disruption	Relevancy Score	Appropriate (Output)
World	Wildfires	+ 0.72	Yes
Politics	Political Crises	+ 1	Yes
Opinion	Mass migration	+ 0.54	No
Tech	Natural Disasters	-0.87	Yes
Science	Nuclear Facilities failure	+ 0.21	No
Health	Infectious disease outbreaks	+ 1	Yes
Business	Port Delays	0.68	Yes
Sports	Offshore Oil Rig Mishaps	0.00	No

The WordNet ontology (Leão et al., 2019) was utilized here for calculating semantic differences between the multiple keywords. The semantic similarity analysis is performed to determine the degree of semantic similarity between the texts. In the fields of NLP, natural language understanding (NLU), and information retrieval, such metrics of text semantic similarity are the most used such as WordNet ontology as a linguistic source because of its wide vocabulary and the explicit definite semantic hierarchy (Zhu et al., 2018, 2020; Gao et al., 2015).

#### Determine the ML task and position the dataset

Classifier Training: The problem of SCD appropriateness to a news article is formulated into two possible outcomes “fake” or “real” as a binary classification. The training set was created from the randomly selected news articles from the dataset. To interpret the training dataset, a supervised learning task was performed with human assistance to make machine learned if a news category “world” is appropriate in supply chain disruption keywords such as “Wildfires”, “Political Crises”, and “Mass migration”. Each keyword pair of news category and SCDs were considered appropriate if the relevancy score is + 0.65. We trained the SCD keywords appropriateness on the selected articles in the training set to predict the appropriateness scores in the test set. We trained numerous frequently used classifiers to train the proposed model, however, because Support Vector Machines (SVM) produced the best results, therefore, we only provide the findings of the SVM classifier in this study.

#### Machine learning technique and interpreting results

Support Vector Machine: We develop a model for each news item by training a binary classifier using two possible outcomes positive (real) and negative (false) news with the appropriateness of SCDs. We chose a binary classification because it is assumed that a responsible user will want to verify the news before spreading it. In order to determine whether the news is real or fake, the user would most likely check online sources (databases/publishers), and in the process, the user may be able to verify or refute the information depending on the source. Subsequently, if the model is trained using a supervised learning technique with only two possible outcomes, the model is forced to make a binary decision, this increases the model’s accuracy significantly. Therefore, the Support Vector Machine (SVM) (Cortes & Vapnik, 1995) classifier was utilized to train the model. SVM is based on a binary classification model, which divides the training samples into two further classes based on multiple support vector hyperplanes in a vector space (Melki et al., 2017; Tharwat, 2019). The supervised learning approach such as SVM is a widely used machine learning method utilizing training examples or datasets to train the model that can be used to solve classification, and regression problems (Melki et al., 2018a, b).

The metrics we utilized for evaluation were; Mean Reciprocal Rank (MRR) (Ghanbari & Shakery, 2019), Precision at 5 (P@5) (Sharma et al., 2020), and Normalized Discounted Cumulative Gain at 5 (NCDG@5) (Alqahtani et al., 2020). A threshold was used for the 5 best matches and the top match was given as the output. These evaluation measures account for the testing accuracy of the constructed model. The comparison between the training set and test set values for MRR, P@5, and NCDG@5 depicted slight differences, this validates the accuracy of the model in the test set with 0.647, 0.656, and 0.511 respectively.

**Table 5:** Model results

Model	MRR	P@5	NCDG@5
Training Set	0.663	0.681	0.594
Test Set	0.647	0.656	0.511

Note: Mean Reciprocal Rank = MRR; Precision at 5 = P@5; Normalized Discounted Cumulative Gain = NCDG@5

### Interview-based validation and proposed model

The impact of fake news on supply chain operations emerged as the first theme from the analysis. When asked about their knowledge and understanding of FNaD, the respondents gave detailed replies. Some argued that it is one of the most harmful aspects of the internet, with the potential to create SCDs. Respondent 4 said that “*Internet information makes us more attentive.*” Respondents 1, 5, 9, 11, and 13 shared the negative impacts of FNaD on quick, routine, and time-consuming decision making: “*Quick decisions based on the inclusion of the FNaD could be a transcendent disaster for any firm’s supply chain*” (Respondent 5). “*if FNaD is included in routine or prolonged decision making, it definitely agonizes the future result*” (Respondent 2), and *“If any decision is based on lies, how can someone expect positive consequences?*” (Respondent 11). Two respondents shared that the purpose of spreading FNaD is to create a specific mindset and narrative in the economy/market to manipulate it: “*Misleading information supposed to build a specific narrative and sentiment in the market, enemies and indirect competitors usually involve in it*” (Respondent 1). The respondents indicated that fake news directly affects operations and has an indirect influence on supply chain operations, contributing to SCDs.

Dealing with FNaD emerged as the second theme of the analysis. The respondents provided numerous options in this regard, suggesting that global communities, social media sites, government, technology, and top management can play key roles in countering FNaD. FNaD should be dealt with smoothly and promptly; otherwise, it will negatively affect supply chain performance: “*As a business community, together we should deal with misleading information and news. Otherwise it could become a scar in business performance*” (Respondent 16). Respondents 12, 15, and 16 mentioned that FNaD should be dealt with as a pandemic like the COVID-19 one. Some respondents mentioned the names of the entities they considered to be primarily responsible for keeping FNaD under control, with Respondents 8, 2, and 11 pointing at the global internet community, social media sites, and the government. On the other hand, respondents 1, 3, 9, 10, 12, and 13 believed that the responsibility of curbing the effects of FNaD on supply chain performance and decision-making should fall on specific industries and businesses. Respondent 13 further explained that *“as a business entity, we need to find a mechanism which guides us that specific news or information is legit or not*”, while Respondent 6 opined that *“in today’s world, if your business isn’t data-driven, then you are definitely living in the jungle.”*

**Fig. 1:**
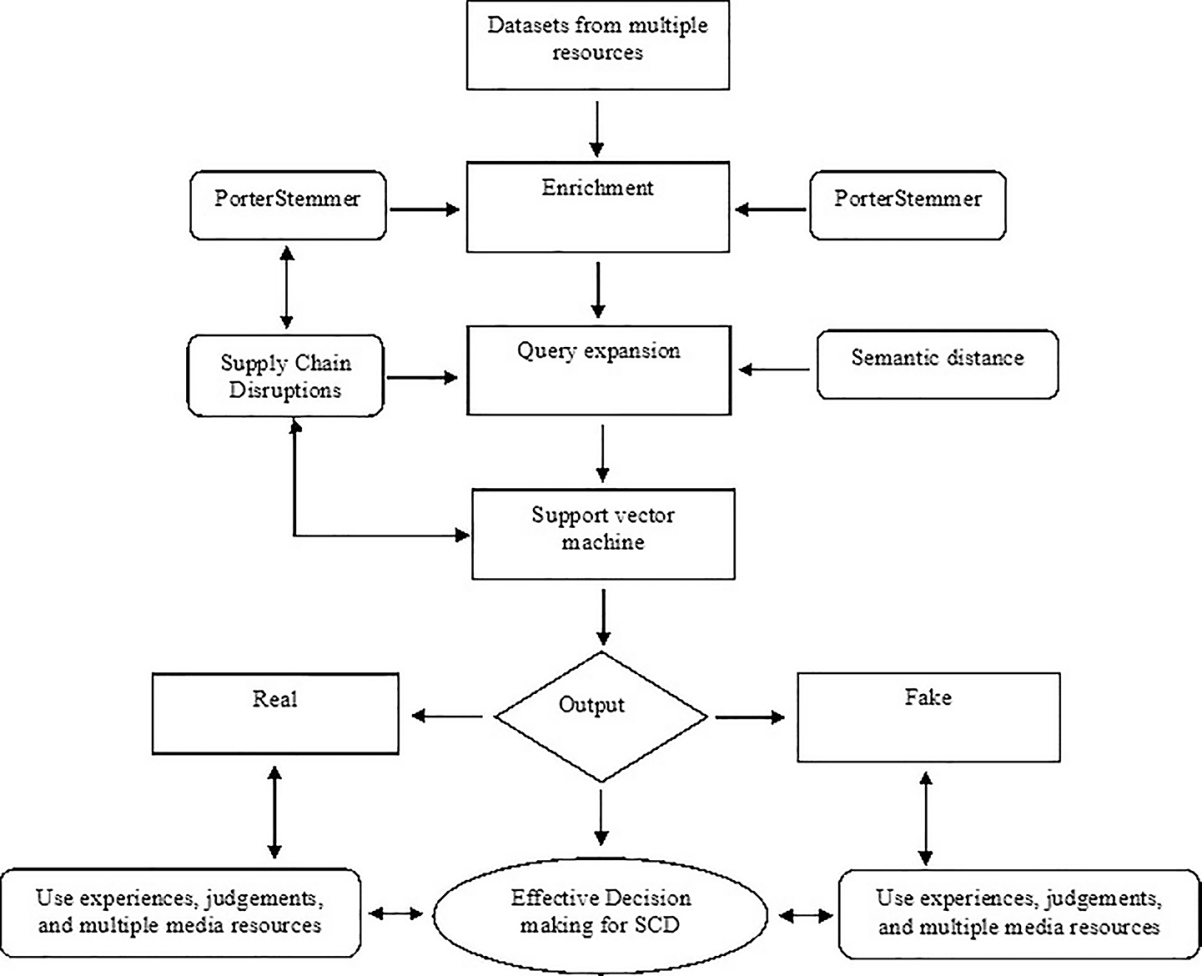
A fake news and disinformation detection model that uses AI and ML

FNaD filtering and counter modeling process suggestions and preparations became the third theme of the analysis. This enriched session contributed many insights into and inputs about countermeasures to FNaD in business and supply chain operations. When we asked Respondent 7 about this, he shared the following Bill Gates quote “*The world won’t care about your self-esteem. The world will expect you to accomplish something before you feel good about yourself*”, and further added *“As a business caretaker, this is my responsibility is to shelter and protect my company from fake news, so that, at the end of the day, I will have no regrets.”* Respondents 3, 5, 7, 10, 14, 15, and 16 suggested that AI will provide solutions suited to control and counter FNaD. Respondent 10 advised that *“Data crawler integration with AI could provide a solution to FNaD”.* Respondent 11 shared a similar thought *“Each government should prepare AI-based processes according to specific society and economy to rectify the impact of fake news, and that process in the form of software should be provided free of cost to businesses”.* The participants also highlighted the importance of using multiple sources to determine whether the news is fake or real, as a single source could be biased or politically driven. Based on the procedure applied for AI, the SVM, and the interview-based validation, we proposed the FNaD detection model shown in Fig. 1, which encapsulates the key findings.

As depicted in Fig. 1, the practical decision-making for SCDs is characterized by the predominant use of experiences, judgments, and multiple media resources. These can be categorized as real and fake news. Data demonstrates that the severity of the fake news impact is prompting businesses to invest in more robust, collaborative, and networked supply chains and should prepare AI-based processes according to specific societies and economies to rectify the impact of fake news. Datasets from multiple sources teach decision-makers about whether the particular news or information is legit or not. The data from multiple sources allows decision makers to apply the machine learning approaches and use artificial intelligence. They can therefore better select the appropriate mechanisms to detect fake and real news.

## Contributions, implications, conclusion, and future research directions

### Contributions and theoretical implications

Our study fills the knowledge gap about SCDs by utilizing AI and ML that assist to act against FNaD affecting supply chain operations. Loureiro et al., (2020) suggested that AI has diverse applications in several industrial domains. Dolgui & Ivanov (2021) hinted that AI could assist in improving resilience against and mitigation of SCDs. We combined a case qualitative method, AI, and SVM in order to reveal how effective decisions could be made within supply chain operations. The extant research advanced our understanding of fake news detection mechanisms using graph and summarization techniques (Kim & Ko, 2021). Furthermore, a recent study proposed an AI-based real-time fake news detection system by conducting a systematic literature review (Gupta et al., 2021). Our study is novel and distinct from the previous ones in that it developed an effective decision-making model for SC firms to avoid any disruptions caused by FNaD. As such, it contributes to the SCDs literature that will be of interest to scholars and practitioners.

Additionally, the study bridges a gap in the literature by providing a practical solution suited to eliminate FNaD in business scenarios affected by SCDs. The scattered and fragmented extant literature had left many questions about FNaD unanswered (Di Domenico & Visentin, 2020). Therefore, the main contribution of our study is to propose an AI- and ML-oriented process capable of flagging/reducing/eliminating FNaD before it reaches decision-makers and of identifying any authentic news and information, thus counteracting SCD-aimed news.

The United Nations (2020) has urged the implementation of actions against misinformation and cybercrime. Edwards et al., (2021) concluded that such ‘digital wildfire’ spreads faster than original and legit news. We propose a process, named FNaD integrated with AI that initiates when news or information is embedded in it. It then begins verification within defined sources (e.g., major newspapers’ websites) and, in the next step, it starts seeking similarities between news or information keywords. Once the AI process reaches a decision, it provides an output by classifying the news item as FNaD (rejection) or real/ authentic news or information (acceptance).

FNaD can be significant determinants of SCDs, as is highlighted in research (Kovács & Sigala, 2021). They adversely influence firms’ operations, import, and export, and alter purchasing behaviors (e.g., Di Domenico et al., 2021; Petit et al., 2019; Wang et al., 2021). The FNaD model shows the ability to control the inclusion of FNaD into firms’ activities. Our study contributes to the management and detection of FNaD in firms’ supply chain operations by proposing and testing a FNaD detection model that uses AI and ML. This model could help to control the potential digital wildfire before it damages firms’ operations. FNaD create unnatural phenomena that interrupt supply chain operations and enhance demand-supply loopholes (e.g., De Chenecey 2018; Dwivedi et al., 2020).

### Managerial and policy implications

Our model detects FNaD early before they can affect firms or managerial decision-making. The current pandemic scenario has turned the attention of managers and governments toward FNaD and their impacts on supply chain operations, economy, and society. On the other hand, with AI and ML becoming an integral part of firms and operations, managers should consider their adoption to deal with FNaD, given their potential to detect and filter them out. Our model is executed and managed based on major local databases and news outlets to support supply chain operations. Should managers wish to integrate adding any further international data and news outlets, they could do so based on their requirements. The implementation of our model would depend on a willing and authoritative IT infrastructure, with even small and medium enterprises being able to invest in its application. We proposed a process capable of detecting and filtering out FNaD. This process protects firms from the impacts of FNaD, enabling managers to engage in decision-making based on legitimate and valid news or information.

From the perspective of specific industries, newsrooms could utilize the FNaD detection model to confirm a news item from different sources. In other words, the FNaD detection model can help in the timely development of a counter-strategy by detecting any fake news before it spreads and causes SCDs. The phenomenon has recently been seen in the context of the COVID-19 pandemic, with people sharing unverified news items on the virus and the side effects of vaccines over social media, thus causing SCDs in vaccine distribution. Moreover, pre-emptive fake news detection can be equally beneficial in avoiding financial market crashes. For government policymakers, the FNaD detection model can be a comprehensive tool to be used during pandemics or similar situations. Governments have been seen to regularly change their decisions, rules, and regulations. Therefore, at the government level, the FNaD detection model can ensure that accurate and on-time legitimate information is received to deal with any economic, social, and health conditions. Another implication for governments pertains to the provision of this process—for free or at a discount—to all business-related entities, especially micro, small, and medium firms. Such a decision would create trust between the government and those entities.

### Conclusion and future research directions

SCDs are problematic for business operations. It is believed that SCDs could cause obstacles due to disinformation. Therefore, we proposed the FNaD model that filters the FNaD by utilizing AI and ML. This model takes help from different sources on internet to verify the received information. It then decides and notifies whether that received news is authentic or not. By using a mixed-method approach, we proposed a way to tackle SCD-creating FNaD using AI- and ML-based techniques. In this regard, future research could, first, focus on more specific FNaD and supply chain operation case studies, such as the detection of FNaD in humanitarian operations using AI and ML approaches. Additionally, they could integrate specific operational performance measures in these approaches, combining them with advanced visual methods. Also, given the fast pace of scientific development, any new and effective algorithm or technique could be used in the proposed model in the future. Furthering, testing the model based on longitudinal studies aimed at exploring and understanding the developments in SCDs linked with FNaD would make it more reliable and refined.
